# Synergistic Cotreatment Potential of Soursop (*Annona muricata* L.) Leaves Extract with Doxorubicin on 4T1 Cells with Antisenescence and Anti-reactive-oxygen-species Properties

**DOI:** 10.22037/ijpr.2020.112485.13788

**Published:** 2021

**Authors:** Irfani Aura Salsabila, Nadzifa Nugraheni, Faradiba Nur Ahlina, Sari Haryanti, Edy Meiyanto

**Affiliations:** a *Cancer Chemoprevention Research Center, Faculty of Pharmacy, Universitas Gadjah Mada, Yogyakarta, Indonesia. *; b *Medicinal Plant and Traditional Medicinal Research and Development Centre, Ministry of Health, Republic of Indonesia, Tawangmangu, Central Java, Indonesia. *; c *Laboratory of Macromolecular Engineering Department of Pharmaceutical Chemistry, Faculty of Pharmacy, Universitas Gadjah Mada Yogyakarta, Indonesia.*

**Keywords:** 4T1 cells, Annona muricata L., Cytotoxic cotreatment, Doxorubicin, Senescence, Reactive oxygen species

## Abstract

*Annona muricata *L. extract (AME) exhibits cytotoxic activities on various types of cancer cells. This study aims to unveil the anticancer activity of AME as a cotreatment agent with doxorubicin (dox) on 4T1 cells and AME’s relation to senescence. AME was obtained by maceration using 96% ethanol. AME was then subjected to qualitative analysis using TLC compared to quercetin (hRf = 75). Spectrophotometry analysis of AME resulted in a total flavonoid content of 2.3% ± 0.05%. Cytotoxic evaluation using the MTT assay revealed that AME showed an IC50 value of 63 µg/mL, while its combination (25 µg/mL) with dox (10 nM) decreased the viability of 4T1 cells to 58 % (CI = 0.15). Flowcytometry using propidium iodide staining confirmed that AME (13 and 25 µg/mL) caused cell cycle arrest in the G1 phase as a single treatment and G2/M arrest in combination with dox. However, by using the dichloro dihydrofluorescein diacetate staining assay, it turned out that AME at concentrations of 13 and 25 µg/mL decreased intracellular reactive oxygen species (ROS) levels both as a single treatment and in combination with dox. *Senescence-associated* *β-galactosidase* assay showed that AME decreased dox-induced senescence. AME alone and in combination with dox (cotreatment) showed cytotoxic effect synergistically on 4T1 cells, but this was not caused by an increase in intracellular ROS levels as well as senescence induction. Therefore, AME showed its potential to be a cotreatment agent with antioxidant property on triple-negative breast cancer cells.

## Introduction

In clinical practice, chemotherapy is one of the most commonly used methods for cancer treatment, and doxorubicin (dox) is still the first-line drug of choice. Unfortunately, a chemotherapeutic agent often leads to a metastasis phenomenon and even failure of therapy (1). This event is often found in the triple-negative breast cancer (TNBC) type of cells, which cannot be treated with specific targeted agents due to the absence of specific receptors. The worse conditions arise when chemotherapeutic agents such as dox induce cancer resistance contributing toto the causes of cancer malignancy. It might be due to the phenomenon of metabolic reprogramming in cancer cells involving glycolysis and the Krebs cycle and/or the mechanism of drug efflux through P-glycoprotein/MDR1 (Pgp) transporters or increased reactive oxygen species (ROS) and free radicals through the redox dox cycle (2, 3). Moreover, common therapeutic agents like dox usually tend to be less specific due to their mechanism involving DNA damage affecting noncancerous cells. These phenomena could be approached through cotreatment using an agent administered together with the main chemotherapeutic agent to enhance the effect of the chemotherapeutic agent or to resolve unwanted adverse effects. Therefore, the exploration of potential cochemotherapeutic (cotreatment) agents is still a challenge in cancer therapy. 

There are several pathways and mechanisms that might explain the anticancer activity of an extract as a cotreatment agent and the main chemotherapeutic agent. These phenomena, however, correlate with the cell cycle and several proliferation pathways. In general, cells carry out metabolic processes to produce energy through the process of aerobic glycolysis, increase the production of fatty acid and undergo glutamine metabolism to conduct proliferation (4). This increased aerobic glycolysis is needed in cell proliferation (5). Anaerobic glycolysis increases in metastatic cancer cells including the TNBC subtype due to abnormal mitochondrial conditions (6). The high metabolism in cancer cells affects the rise of intracellular ROS levels compared normal cells. The high production of ROS in TNBC cells is also supported by the high metabolism of an endogenous or exogenous aldehyde with the enzyme aldehyde dehydrogenase (7). However, in cancer cells, levels of ROS that exceed the threshold can direct cells to death or apoptosis (8). Some natural ingredients such as *Caesalpinia sappan* L. and other Indonesia herbs (9, 10), *Piper nigrum* L. (11), and *Carica papaya* (12) are even known to affect intracellular ROS levels and interfere with cancer cell proliferation. In contrast, sublevels of ROS may induce cell proliferation, and cell migration and metastasis. Thus, intracellular ROS levels have a very important role in determining the life of cancer cells. Therefore, studies of intracellular ROS pathways, particularly in the TNBC type of cells, are important as a basis for developing more targeted cancer therapies (13, 14).

Soursop (*Annona muricata* L.) leaves extract has long been studied and proven to contain phenolic compounds, flavonoids, and acetogenin, which have effects on the physiological and cellular conditions of cancer cells (15). *Annona muricata* L. extract (AME) induces apoptosis and demonstrates antiproliferative effects associated with a cell cycle arrest in the G1 phase of several types of colon cancer cells (16). In addition to its cytotoxic activity, soursop leaves extract also inhibits migration, induces apoptosis, and causes cell cycle arrest in several breast cancer cells (17). A comprehensive review of soursop shows cytotoxic activities of soursop leaves extract against various types of breast cancer cells, but reports of its cytotoxic effects on 4T1, a TNBC model, can still be explored more (15). The 4T1 is a cancer cell line derived from a spontaneously arising BALB/c mammary tumor. These cells were characterized with the highly metastatic activity (18, 19). 4T1 cells even more suitable as a model for TNBC due to the low to absence of estrogen receptor (ER), progesterone receptor (PR), and human epithelial growth receptors (HER-2) (20). These conditions were alike with the condition of TNBC in general. Consequently, the condition caused a non-responsive effect for TNBC towards convenient treatments used for metastatic breast cancer. The absences of these receptors are which therefore requires special treatment approaches (21). The activities of *Annona muricata* L. sparked the possibilities to develop this plant as a cotreatment agent with dox. Although it was already known that soursop leaves extract and its active compounds express a cytotoxic effect on various cancer cells, the target mechanisms remain unclear. Thus, the focus of this study is to trace the mechanisms possibly involved in the anticancer effects of AME and its combination with dox in relation to ROS generation.

## Experimental

*AME preparation*

*Annona muricata *L. leaves were obtained and determined from UPT Materia Medica Batu, Malang, with the herbarium code: 074/164A/102.7/2018. The leaves were then powdered and sifted. The powder was weighed for 70 g then macerated with 96% ethanol. Maceration was conducted for 24 h, and then remaceration was conducted after 48 h. Then, it was evaporated using a rotary evaporator. The yield of extract obtained as much as10.10%.

*Cell culture*

The 4T1 breast cancer cells (ATCC® CRL-2359) from Professor Masashi Kawaichi, Nara Institute of Science and with 10% fetal bovine serum and 1% penicillin– streptomycin under standard conditions (37 °C; 5% CO2).

*MTT assay*

MTT assay was carried out based on Mosmann,1982 (22, 9). In this study, 2.5 × 10^4^ 4T1 cells were plated onto every well of 96-well plates and treated with a series of concentrations of AME and in combination with dox (Sigma) for 24 h. MTT reagent (Sigma) was then added, and incubation was done for 4 h. Colored formazan crystals were formed, and then the absorbances were measured under a microplate reader after being diluted in sodium dodecyl sulfate solution (Bio-Rad) (*λ* = 595 nm).

*Direct counting assay*

The cells (1 × 10^4^) were seeded into every well of 6-well plates. The cells were incubated for 24 h in the DMEM. The test solution in the form of AME and dox dissolved in DMSO and each media as much as 500 µL was added to the well and then reincubated for 24 h. The cells were then harvested in 1 mL of culture media from each well. Then, the suspension was taken (10 µL) and resuspended with 10 µL of trypan blue. After that, the cells were directly counted using a hemocytometer under an inverted microscope.

*Cell cycle analysis*

4T1 cells (1 × 10^5 ^cells/well) were distributed onto a 6-well plate and incubated for 48 h. Afterward, cells were treated with various concentrations of AME along with dox; both the single solution and its combination were then incubated for 24 h. Both supernatant and pellet were collected from the very beginning sequence, then finally pelleted at 2,000 rpm for 3 min, then flushed with phosphate-buffered saline (PBS).With the cell’s pellets, 50 µg/mL of propidium iodide was suspended in the presence of 100-µg/mL RNase and triton-x solution and incubated for 10 min at 37 ºC. The cell cycle distribution was analyzed using the BD Accuri C6 flowcytometer, while the percentages of the cell population at each phase of the cell cycle were determined by the BD Accuri C6 software.

*Intracellular ROS analysis*

Intracellular ROS level measurement was adopted from Wu and Yotnda, 2011 (23). In this study, we seeded 4T1 cells (8 × 10^4^) into a 24-well plate and incubated them for 24 h. Then, each well was harvested using trypsin–EDTA (75 µL) for each well. Thirty-two microliters of a supplemented buffer consisting of PBS and 10% FBS was added to deactivate trypsin. Then, we performed cell staining with 25-µM dichloro dihydrofluorescein diacetate (DCFDA) by adding 3.75 µL of 2-mM DCFDA to each microtube. Incubation was done for 4 h at 37 °C. The ROS level was then scanned using the BD Accuri C-6 flowcytometer.

*SA-β Gal senescence-based assay*

Evaluation of senescence induction was conducted based on Debacq–Chainiaux *et al.*, 2009 (24). In this study, 4T1 cells (2 × 10^5^ cells/well) were plated into each well of 6-well plates and incubated for 24 h. Cells were then flushed with PBS twice. Fixation buffer was then added, then the cells were flushed with PBS once. Afterward, 1–2 mL of X-Gal solution was added. Then, the cells were incubated at 37 °C. Under a microscope (CKX41 Olympus), cells were observed after 72-h incubation. Senescent cells will appear as colored blue cells due to their galactosidase content.

*TLC and determination of total flavonoid contents*

A certain amount of extract was weighed and dissolved in 96% ethanol. The mobile phase used is a mixture of butanol: acetic acid: water (5:4:1). Comparative compounds of quercetin were used from the flavonoid group. The results of the TLC test were then observed under 254-nm and 360-nm UV light before and after being evaporated with NH3, respectively. The spots of AME were then calculated to be compared with quercetin and quantified with hRf.

Determination of total flavonoid levels was carried out by the spectrophotometry method. AME was weighed and then dissolved in methanol with a concentration of 100 mg/mL. Then, 33.6 µL of the solution was dissolved to a volume of 1.2 mL so that the sample solution was obtained with a concentration of 2.8 mg/mL. From the sample solution, 500 μL was taken, and then 1.5 mL of methanol was added. Then, 100 μL of 10% AlCl3, 100 μL of sodium acetate, and 2.8 mL of distilled water were added consecutively. The solution was incubated for 30 min, and the absorbance was scanned at 436 nm. Flavonoid levels were obtained from the quercetin standard curve regression equation.

*Statistical Analysis*

Data were accorded as mean ± SD, followed by statistical analysis using Student’s *t*-test. The *p*-values (^*^p < 0.05; ^**^p < 0.01) were included in every figure attached.

## Results

*Cytotoxic effect of AME on 4T1 cells*

**Figure 1:**
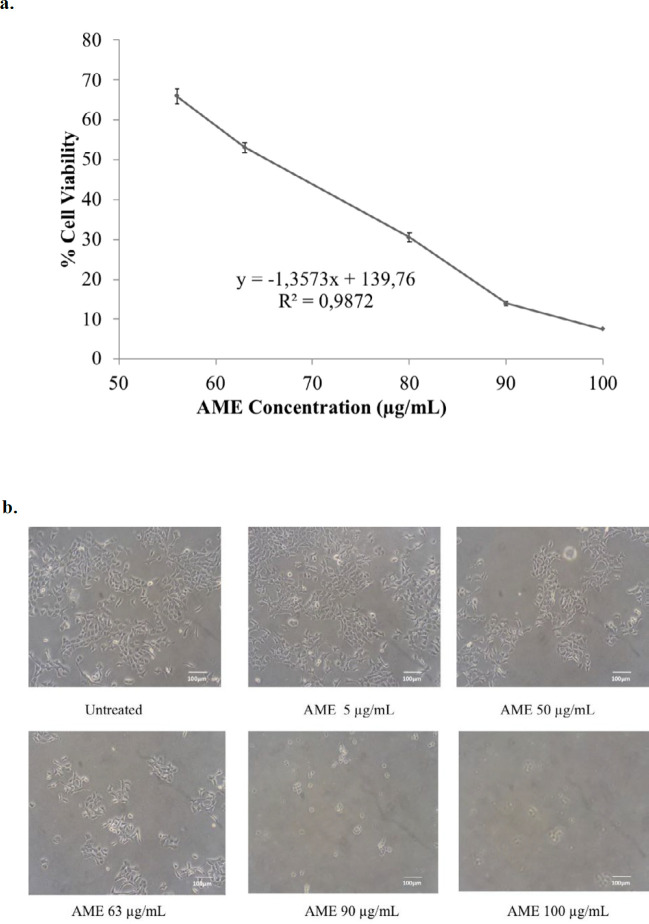
Cytotoxic effect of AME on 4T1 cells. 4T1 cells (5 × 10^4^ cells/mL) were treated with AME for 24 h and then subjected to MTT assay. The viable cells were then quantified using a 450-nm ELISA reader. (a) Percentage of 4T1 cells viability. The viable cells were calculated in accordance with the analysis procedure (*p *< 0.05). (b) Cells’ appearance after being treated with AME under an inverted microscope with a 100x magnification

**Figure 2:**
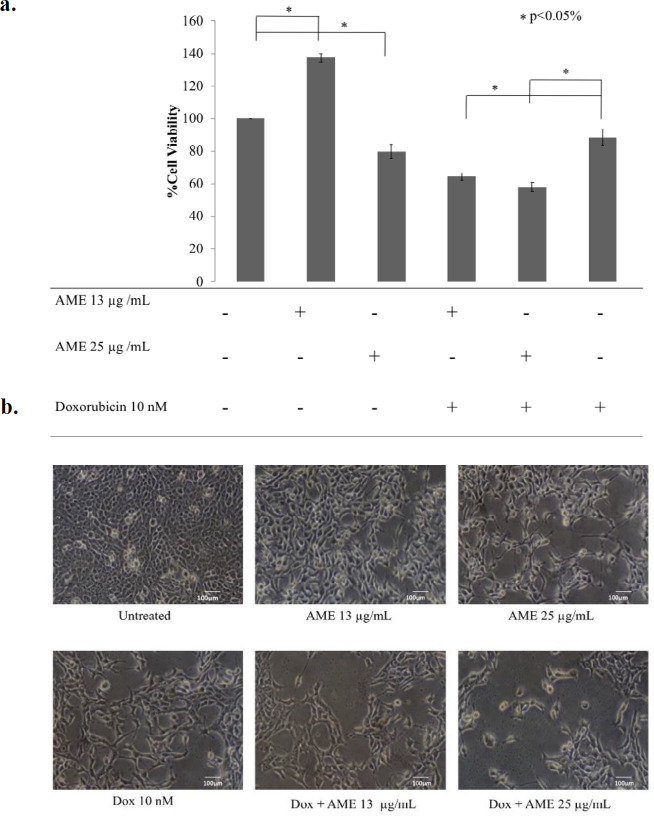
Cytotoxicity of AME in combination with dox. 4T1 cells (5 × 10^4^ cells/mL) were treated with 13 μg/mL and 25 μg/mL of AME, and its combination with dox (10 nM) for 24 hwas then subjected to counting with trypan blue. (a) The percentage of 4T1 cells viability.The viable cells were calculated in accordance with the analysis procedure (*p *< 0.05). (b) The cells’ appearance after being treated with AME under an inverted microscope with a 100× magnification

Soursop leaf extract possessed a cytotoxic effect on 4T1 cells in a dose-dependent manner. The IC50 value of AME in 4T1 cells was 63 µg/mL (Figure 1). This result indicates a strong cytotoxic effect (21). On the other hand, the combination of AME at concentrations of 13 and 25 µg/mL with 10-nM dox caused a strong antiproliferative effect which was shown by lower cell viabilities as much as 64% (CI = 0.07) and 58% (CI = 0.15), respectively, compared to single treatment with dox (*p *< 0.01) (Figure 2). Therefore, if AME is used with dox, AME can be developed as a cotreatment agent. Although it was confirmed that AME possessed cytotoxic properties toward 4T1 cells, the mechanism causing this effect remains unclear. However, several mechanisms include elevation of intracellular ROS levels, induction of senescence, apoptosis, and cell cycle arrest that might explain the cytotoxic phenomenon.

*Effect of AME on cell cycle profile*

**Figure 3:**
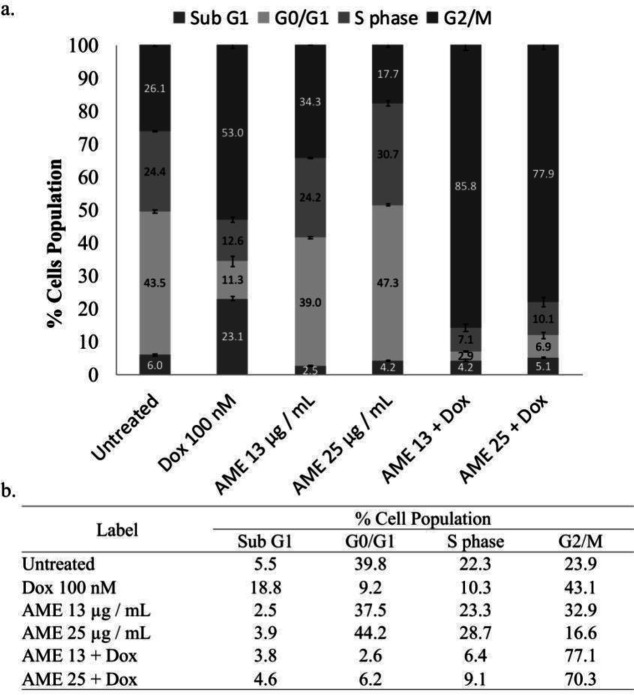
Cell cycle distribution effect of AME alone and in combination with dox. (a) 4T1 cells (3 × 10^3^ cells/mL) were treated with 12.5 and 25 µg/mL of AME alone and in combination with 100-nM dox for 24 h and subjected to cell cycle analysis under PI staining with flow cytometry (n = 3). (b) The summarization of % cell population of 4T1 cells for each phase

To understand the effect of AME on the cell cycle progression, we conducted flow cytometry analysis in AME alone and in AME in combination with dox. We found that AME (25 µg/mL) as a single treatment induced significant cell cycle arrest in the G1 phase on 4T1 cells, while its combination with dox (100 nM) resulted in accumulation of the G2/M phase. Dox alone also induced accumulation of the G2/M phase, but the combination of both increased accumulation significantly (Figure 3). The treatments were suspected of causing cell cycle arrest that may correlate with the senescence phenomenon or ROS modulation.

*AME decreases senescence on 4T1 cells*

**Figure 4:**
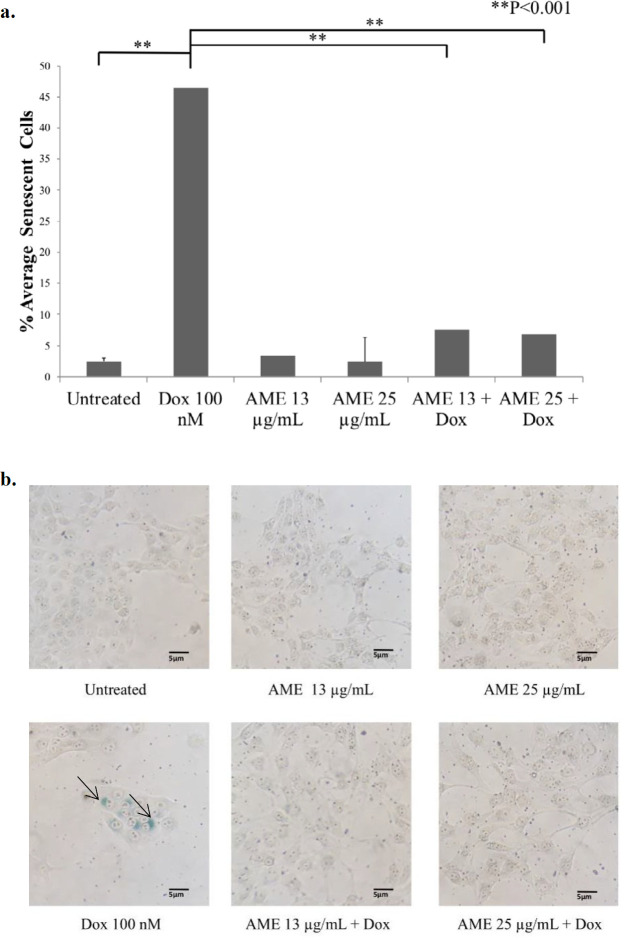
Effects of AME on 4T1 cells *in-vitro*. (a) 4T1 cells (5 × 10^5^ cells/mL) were treated with Annona muricata (12.5 μg/mL and 25 μg/mL) alone and in combination with dox (100 nM) for 1, 2, and 3 days and subjected to β-galactosidase staining. As a positive control, cells were treated with dox (100 nM) for 3 days. The percentages of senescent cells (β-galactosidase-positive cells) were calculated (n = 3). (b) The senescence induced cells’ appearance after being treated with AME and dox under an inverted microscope with a 100× magnification

One of the possible mechanisms of AME as an anticancer agent is senescence induction that is believed to cause termination of a cell’s proliferative ability. To unveil this possibility, we then examined the effect of AME on 4T1 cells with a β-galactosidase-based assay. Dox was used not only as of the chemotherapeutic model but also as a senescence-inducing agent (26). The results indicated that the untreated cells were still found to be senescence positive by 3%, while the dox-treated cells increased senescence significantly (*p *< 0.01) by 26%. However, treatment with AME did not change the senescence, and also, the combination of AME and 100-nM dox decreased the senescence-positive cells compared to dox alone (Figure 4). Therefore, it might be inferred that the cytotoxic properties of AME alone and in combination with dox do not correlate with the senescence evidence. In some cases, senescence evidence is correlated with ROS generation in the cell.

*The effect of AME on the intracellular ROS levels of 4T1 cells*

**Figure 5:**
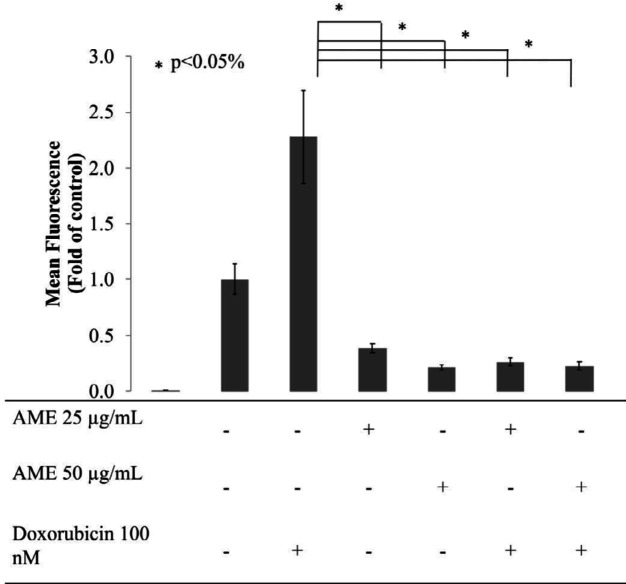
Effect of AME on the intracellular ROS levels of 4T1 cells. Treatment with AME alone and in combination with dox decrease intracellular ROS levels. Cells (8 × 10^5^ cells/mL) were treated with AME (25 and 50 μM), dox (100 nM), and both for 4 h and subjected to ROS detection analysis using flow cytometry. AME decreased the ROS levels in 4T1 cells after the 4-h treatment

Intracellular ROS level is intriguing and reflects the proliferative status of cells. At sublevels, intracellular ROS acts as a second messenger that induces cell proliferation. ROS can directly stimulate the phosphorylation of mitogen-activated protein kinase and extracellular signal-regulated kinase (ERK) (27). As a consequence, cell division and proliferation occur on a large scale, causing gene mutations and metabolic changes. This causes an increase in the level of intracellular ROS followed by an increase in the expression of antioxidant enzymes (28). However, precisely because of this condition, cancer cells produce ROS and antioxidants at a high level, and cancer cells become more susceptible to changes in ROS levels. A very high level of ROS can induce an intrinsic apoptotic pathway. In addition to being used as a TNBC chemotherapy model, in this study, dox was used as an inducer of the production of intracellular ROS primarily in DCFDA staining assay. In human colorectal cancer cells DLD-1, dox causes an imbalance of antioxidants–oxidants through induction of oxidative stress. In general, cancer cells, dox increases the production of excessive intracellular ROS (29). Therefore, in this study, 4T1 cells were treated with AME at a single concentration of 25 μg/mL and its combination with 100-nM dox. As a result, dox induced the production of ROS in 4T1 cells significantly compared to that in the untreated cell, while both single AME treatment and its combination with dox caused a significant decrease in the levels of ROS in 4T1 cells (*p *< 0.05) (Figure 5).

*AME flavonoid content*

**Figure 6:**
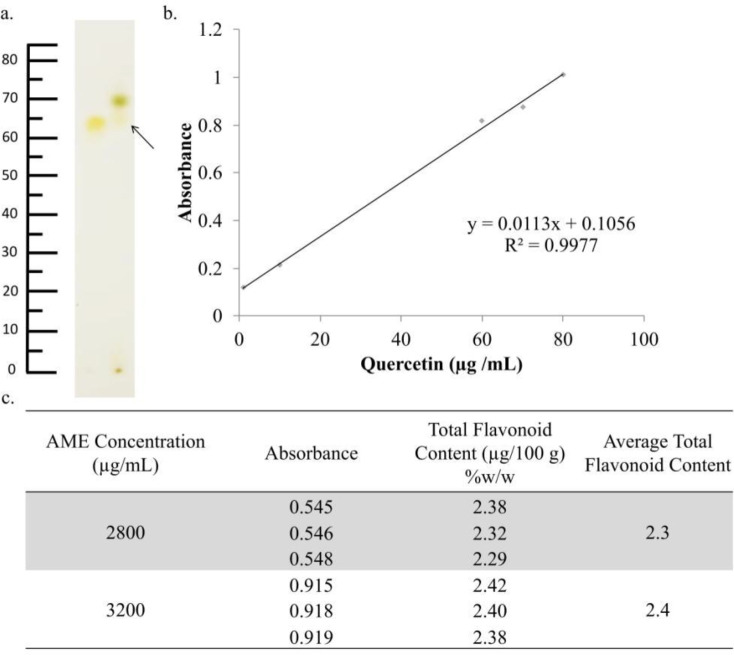
Identification of the chemical content of AME. AME was identified using TLC, while the total flavonoids were determined using spectrophotometry. (a) TLC chromatogram of AME (2) and quercetin (1) showed relatively similar spots between quercetin as standard and AME with an Rf value 0.70. (b) The regression equation obtained from quercetin standard measurement (c) The total flavonoid determination indicated that the total flavonoid content of AME was 2.30% ± 0.05%.

The treatment of AME resulted in a decreasing level of senescence ROS in 4T1 cells. These effects were possibly caused by the existence of antioxidant compounds in AME. Most of these compounds are polyphenolic compounds and were classified as flavonoid. using the TLC method, AME showed a similar profile with quercetin as the flavonoid reference compound (hRf = 75) (Figure 6a). This result indicated that AME contains flavonoid compounds (15). These results were confirmed by the determination of total flavonoids with the same reference compound, quercetin. Determination was carried out at a wavelength of 436 nm (30). The total flavonoids obtained were as much as 2.3% ± 0.05% (Figure 6c). This result is not much different when compared with previous studies, which stated that AME has a total flavonoid level of 2.10% ± 0.24%. Compared to other extracts such as *Zea mays* L. peel extract with a total flavonoid content of 6.94% ± 0.51% (31), that of *Alpinia galangal* L. amounted to 1.378% (32) and that of *Citrus hystrix* peel amounted to 11.47% (33). The total flavonoids contained in AME were likely to be quite a lot.

## Discussion

AME was already known for its cytotoxicity toward various types of cancer cells. AME toward HCT-116 and HT-29 cells gave IC50 values of 11.437 ± 1.87 µg/mL and 8.987 ± 1.24 µg/mL, respectively,while toward MDA-MB-231 TNBC type, AME had a cytotoxic effect with an IC50 value greater than 100 µg/mL (34, 35). Meanwhile, in MCF-7 cells representing ER+ breast cancer cells, AME had a cytotoxic effect with an IC50 of 6.2 µg/mL (36). In this study, once again, AME showed its potential as an anticancer agent by its cytotoxic effect toward 4T1 cells with an IC50 value of 63 ± 6.8 µg/mL. However, the differences in IC50 values are very likely to occur due to different sources of the plants and the differences in the features and molecular characteristics of the cell targets. These effects may be caused by several compounds contained in AME, such as acetogenin, annomuricin, annonacin, and flavonoid compounds (37).

In developing the natural product for anticancer, it is interesting to consider exploring the standardized extract to make it simpler to be used in clinical application. In this concern, AME showed a cytotoxic effect on 4T1 cells not only as a single treatment but also in combination with dox, with significant evidence considered to be a synergistic property. This increasing cytotoxic effect with dox likely correlates with the effect on cell cycle arrest in G2/M. In this experiment, we did not find an increase in the sub-G1 phase that indicates the apoptosis evidence (Table 1). This phenomenon may occur due to the short observation time (24 h) where cells still undergo arrest before facing apoptosis. The synergistic effect of AME with

dox is an important note that AME shows the potential to be developed as a cochemotherapeutic agent.

Dox is a chemotherapeutic agent that is commonly used to cure TNBC. Its cytotoxicity to cancer cells is suggested to correlate with senescence induction, but it is not selective. In our TNBC cells model, 4T1 cells, dox also induced senescence which may confirm the cytotoxic activity of dox to this cell line. However, in contrast, AME did not show a significant increase in senescence in 4T1 cells and exhibited a decrease in the senescence effect of dox instead. Moreover, AME and its combination with dox significantly decreased the levels of ROS in 4T1 cells. 4T1 cells express relatively high enzymes metabolizing ROS (38). Furthermore, the metastatic cancer cells also have a different expressions to ROS-related protein depending on the site of the cancer cells affected (10). However, this is an exciting finding because it indicates that the cytotoxic effects of AME are not associated with an increase in intracellular ROS levels. It causes a significant decrease in ROS levels instead. This phenomenon may occur due to the vast number of flavonoid compounds in AME (Table 2). Flavonoid compounds are known for their antioxidant activity due to their compound structure which has many phenolic residues bound to the generic carbon ring framework (39).

There are several mechanisms of radical scavenging that might explain the findings. One of them is the H+ atom of the phenolic group of flavonoid compounds to scavenge radical electrons (23). Flavonoids are oxidized by radicals, producing more stable and less reactive radicals. In other words, flavonoids stabilize ROS by reacting with radical reactive compounds (40). Many levels of flavonoids from AME allow antioxidant activity, which causes a decrease in intracellular ROS levels. Sufficient evidence shows that using a single acetogenin or a combination of several acetogenins has a toxic effect (41).

From this discovery, AME’s possible cytotoxic mechanisms are caused by acetogenin involving inhibition of the mitochondrial complex to result in hypoxia and the failure of ATP production in cancer cells. The ROS-decreasing property of the antioxidant-rich natural product is an exciting focus in anticancer studies, contrasting with the ROS-increasing property. The cytotoxic effect of AME in relation to ROS depletion is an interesting phenomenon to be a special note as an advantage of this plant. However, we should consider the other possibilities of its mechanisms as anticancer that may be nonselective.

One of the acetogenin compounds found in this plant, namely, Annomuricin E, down-regulates the Bcl-2 protein and increases the regulation of Bax expression leading to apoptosis (34). In addition, it was suggested that on pancreatic cancer cells, AME suppresses the phosphorylation of key molecules involved in the ERK and PI3K/Akt pathways resulting in the inhibition of proliferation and survival of pancreatic cancer cells (42). These possibilities may occur to contribute to cytotoxicity, but these target mechanisms also exist in non cancerous and even healthy cells in normal physiological processes. Therefore, we still need more studies about the characteristics of the cytotoxic effect of AME to be used as a cotreatment agent in breast cancer therapy.

**Table 1:** Total flavonoid content of AME

**AME Concentration (µg/mL)**	**Absorbance**	**Total Flavonoid Content (µg/100 g) (%) (** ^w^ **/**_w_ **)**	**Average Total Flavonoid Content (µg/100 g) (%) (** ^w^ **/**_w_ **)**
2800	0.545	2.38	2.3
	0.546	2.39	
	0.548	2.29	
3200	0.915	2.42	2.4
	0.918	2.40	
	0.919	2.38	

**Table 2:** 4T1 Cells Distribution in Cell Cycle

**Label**	**Cell Population (%)**			
	**Sub G1**	**G0/G1**	**S**	**G2/M**
Untreated	5.5	39.8	22.3	23.9
Dox 100 nM	18.8	9.2	10.3	43.1
AME 13 µg / mL	2.5	37.5	23.3	32.9
AME 25 µg / mL	3.9	44.2	28.7	16.6
AME 13 + Dox	3.8	2.6	6.4	77.1
AME 25 + Dox	4.6	6.2	9.1	70.3

## Conclusion

Overall, from this study, AME showed its synergistic cotreatment potential with dox on 4T1 cells that may correlate with the decreasing effect on senescence and ROS levels.
